# Spontaneous mutations in *Streptococcus pyogenes* isolates from streptococcal toxic shock syndrome patients play roles in virulence

**DOI:** 10.1038/srep28761

**Published:** 2016-06-28

**Authors:** Tadayoshi Ikebe, Takayuki Matsumura, Hisako Nihonmatsu, Hitomi Ohya, Rumi Okuno, Chieko Mitsui, Ryuji Kawahara, Mitsuhiro Kameyama, Mari Sasaki, Naomi Shimada, Manabu Ato, Makoto Ohnishi

**Affiliations:** 1 National Institute of Infectious Diseases, Department of Bacteriology I, Tokyo, 162-8640, Japan; 2 National Institute of Infectious Diseases, Department of Immunology, Tokyo, 162-8640, Japan; 3 Fukushima Institute of Public Health, Department of Microbiology, Fukushima, 960-8560, Japan; 4 Kanagawa Prefectural Institute of Public Health, Division of Microbiology, Kanagawa, 253-0087, Japan; 5 Tokyo Metropolitan Institute of Public Health, Department of Microbiology, Tokyo, 169-0073, Japan; 6 Toyama Institute of Health, Department of Bacteriology, Toyama, 939-0363, Japan; 7 Osaka Prefectural Institute of Public Health, Department of Infectious Diseases, Osaka, 537-0025, Japan; 8 Yamaguchi Prefectural Institute of Public Health and Environment, Department of Public Health Sciences, Yamaguchi, 753-0821, Japan; 9 Oita Prefectural Institute of Health and Environment, Laboratory of Microbiology, Oita, 870-1117, Japan; 10 Saitama Institute of Public Health, Laboratory of Clinical Microbiology, Saitama, 355-0133, Japan

## Abstract

*Streptococcus pyogenes* (group A *Streptococcus*; GAS) is a widespread human pathogen and causes streptococcal toxic shock syndrome (STSS). STSS isolates have been previously shown to have high frequency mutations in the *csrS/csrR* (*covS/covR*) and/or *rgg* (*ropB*) genes, which are negative regulators of virulence. However, these mutations were found at somewhat low frequencies in *emm1*-genotyped isolates, the most prevalent STSS genotype. In this study, we sought to detect causal mutations of enhanced virulence in *emm1* isolates lacking mutation(s) in the *csrS/csrR* and *rgg* genes. Three mutations associated with elevated virulence were found in the *sic* (a virulence gene) promoter, the *csrR* promoter, and the *rocA* gene (a *csrR* positive regulator). *In vivo* contribution of the *sic* promoter and *rocA* mutations to pathogenicity and lethality was confirmed in a GAS mouse model. Frequency of the *sic* promoter mutation was significantly higher in STSS *emm1* isolates than in non-invasive STSS isolates; the *rocA* gene mutation frequency was not significantly different among STSS and non-STSS isolates. STSS *emm1* isolates possessed a high frequency mutation in the *sic* promoter. Thus, this mutation may play a role in the dynamics of virulence and STSS pathogenesis.

Group A *Streptococcus* (GAS) is one of the most common human pathogens and causes various infections, ranging from uncomplicated pharyngitis and skin infections to severe and even life-threatening manifestations, such as necrotizing fasciitis (NF) and bacteremia^1^. Streptococcal toxic shock syndrome (STSS) is a severe invasive infection recently characterized by the sudden onset of shock and multi-organ failure and has a high mortality rate, ranging from 30% to 70%^2^.

STSS isolates were previously shown in a study to have high frequency mutations in the GAS two component regulatory system genes, *csrS* (*covS*) and *csrR* (*covR*), and/or the *rgg* gene, all of which are negative regulators of virulence. STSS isolates with these mutations elevated the expression of various virulence genes, and were associated with *in vitro* escape from host defenses and *in vivo* lethality, as seen using a mouse STSS model. These mutations were thus regarded as responsible factors for STSS^3^. In this study of clinical STSS isolates, the frequency of these mutations in *emm*-genotyped isolates, excluding the prevalent *emm1* genotype, was 71.4% (65/91 isolates), whereas that of the *emm1*-genotyped isolates was 39.7% (29/73). Additionally, 44 *emm1* STSS isolates did not have any mutations in these genes^3^. Thus, these mutations were significantly found in *emm* genotypes other than *emm1* (*p* = 0.000045 by Χ^2^ analysis). Therefore, we hypothesized that there are other mutation(s) in these 44 *emm1*-genotyped STSS isolates responsible for enhanced virulence. In this study, we examined this hypothesis by identifying the STSS *emm1* isolates that significantly elevated the expression of important virulence genes such as *scpA*, *sic, sda1, nga*, and *ska* as compared to non-invasive or non-STSS isolates to detect the novel mutations underpinning this enhanced virulence and demonstrate pathogenicity of these mutations in a GAS mouse model. Such insights will shed light on the mechanisms underlying pathogenicity in STSS *emm1*-genotyped isolates.

## Results

### Selection of *emm1* STSS isolates with significantly elevated expression of key virulence genes

For our study, we conducted quantitative RT-PCR from 44 *emm1* STSS isolates lacking mutations in the *csrS*, *csrR*, and *rgg* genes and 3 *emm1* non-invasive isolates (negative controls) to assess the expression of five virulence genes, *scpA, sic, sda1, nga*, and *ska*, known to be elevated in invasive strains^4^. We selected isolates with expression levels >10-fold in at least one virulence gene as compared to controls (Table 1). Ten isolates met this criterion, with four showing elevated expression in all five genes and six with elevation only in the *sic* gene. We then systematically set out to identify the mutations responsible for enhanced expression of these virulence genes.

### Promoter mutation of *csrR/csrS* operon

**Figure 1:**
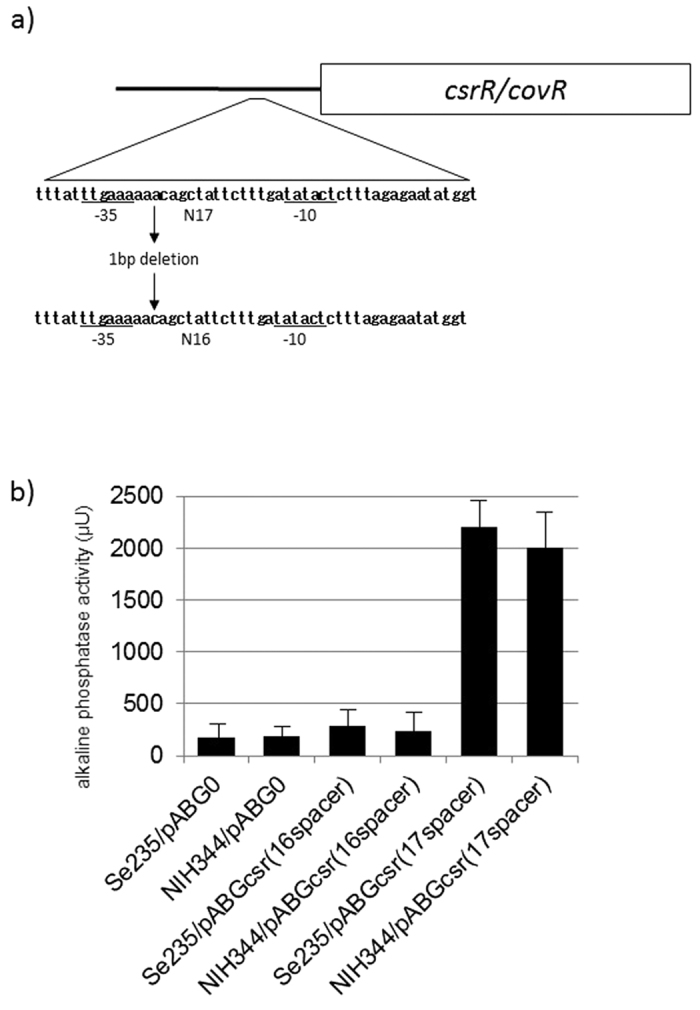
The sequence of *csrR* promoter and *csrR* promoter activity. (**a**) *csrR* promoter sequence in which 1 bp was deleted in the promoter region of NIH342 and NIH344 STSS isolates, particularly in the putative −10 and −35 regions (underlined). Number next to N indicates the length of spacer between −10 and −35 DNA elements. Box indicates the *csrR/covR* gene. (**b**) Transcriptional level of the *csrR* gene in a non-invasive isolate (Se235) and a STSS isolate with the 1-bp deletion in the promoter region (NIH344). To measure the transcriptional levels of *csrR* genes, we used *csrR*-*phoZF* transcriptional fusion plasmids to assess alkaline phosphatase activity. Mean activity ± SD of each strain is shown for three independent experiments. Error bars show the standard deviation (SD).

We sequenced the promoter regions of *csrR/csrS* and *rgg* genes. In the *emm1* STSS NIH342 and NIH344 isolates, a 1-bp deletion in the *csrR/csrS* promoter was observed when compared to the *csrR/csrS* promoter of the non-STSS Se235 isolate (aaac to aac) (Fig. 1a). This deletion was positioned between the −35 and −10 elements of the promoter as determined in Gusa & Scott (2005)^5^ and shortened the spacer length between the −35 and −10 elements from 17 bp to 16 bp (Fig. 1a). We hypothesized that this deletion inhibited expression of the *csrR/csrS* promoter, thus resulting in enhanced virulence. To measure the transcriptional level of the *csrR* gene, we introduced the *csrR*-*phoZF* transcriptional fusion plasmid either with the deletion (pABGcsr(16spacer); as derived from NIH344) or without the deletion (pABGcsr(17spacer); as derived from Se235) into NIH344 and Se235 isolates and measured alkaline phosphatase activity. Additionally, we introduced pABG0 plasmids into these isolates. Isolates harboring pABGcsr(16spacer) had lower promoter activity than isolates harboring pABGcsr(17spacer) (Fig. 1b). Thus, the 1-bp deletion in the *csrR/csrS* promoter of STSS *emm1* isolates NIH342 and NIH344 resulted in decreased expression of *csrR/csrS* genes.

### Promoter mutation of *sic* gene

**Figure 2:**
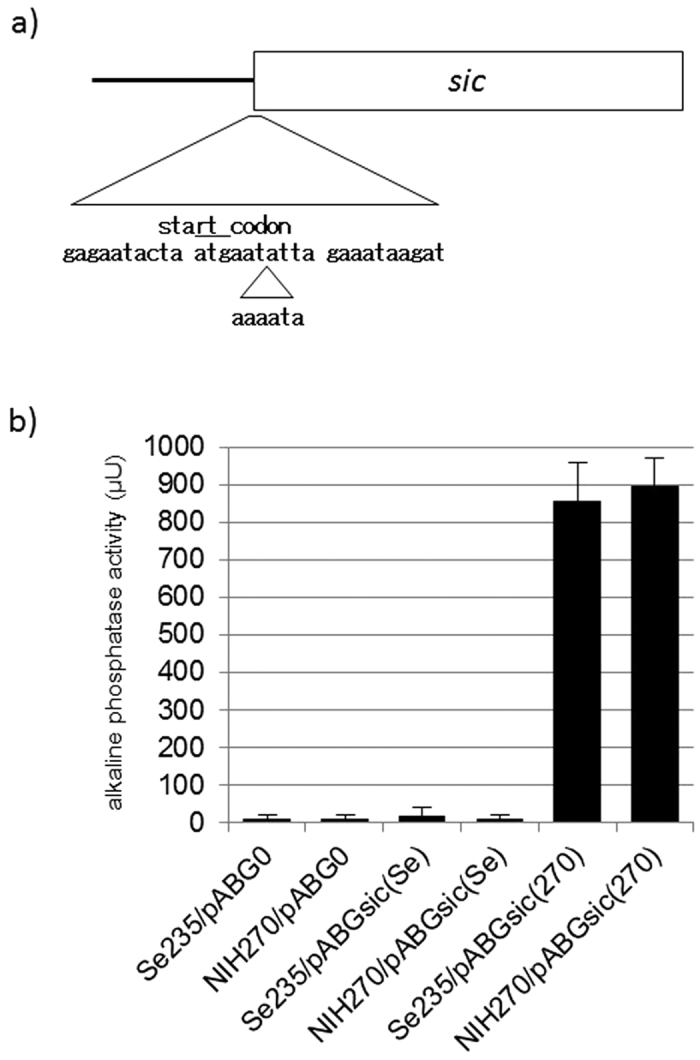
The sequence of *sic* promoter and *sic* promoter activity. (**a**) Sic promoter sequence. A 6-bp (aaaata) sequence was inserted in STSS isolates (such as NIH270) near the start codon (next to atgaat). The start codon (atg) is indicated by the upper line. Box indicates the *sic* gene. (**b**) Transcriptional level of the *sic* gene in each strain, Se235 and NIH270. To measure the transcriptional levels of *sic* genes, we used *sic*-*phoZF* transcriptional fusion plasmids and assessed alkaline phosphatase activity. Mean activity ± SD of each strain is shown for three independent experiments. Error bars show SD.

Six *emm1* STSS isolates (NIH135, NIH150, NIH185, NIH270, NIH392, and NIH436) only elevated the expression of the *sic* gene, a virulence gene encoding the extracellular streptococcal inhibitor of complement (Table 1). We hypothesized this was due to a mutation in the *sic* promoter and thus sequenced the region. We found a 6-bp insertion (aaaata) in all six isolates (Fig. 2a). We discerned this insertion’s effect on *sic* transcription via alkaline phosphatase reporter assays. We either introduced the *sic*-*phoZF* transcriptional fusion plasmid pABGsic(270), containing the *sic* promoter derived from NIH270, or the pABGsic(Se) plasmid, containing the *sic* promoter derived from Se235, into NIH270 and Se235 isolates, respectively (Table 2) and measured alkaline phosphatase activity. Strains harboring pABGsic(270), and thus the insertion, exhibited higher activity than strains harboring pABGsic(Se), which lacked the insertion (Fig. 2b). Therefore, the increase of *sic* expression is due to this 6-bp insertion found in the six *emm1* STSS isolates.

### Mutations of the *rocA* and *scaR* regulator genes

**Figure 3:**
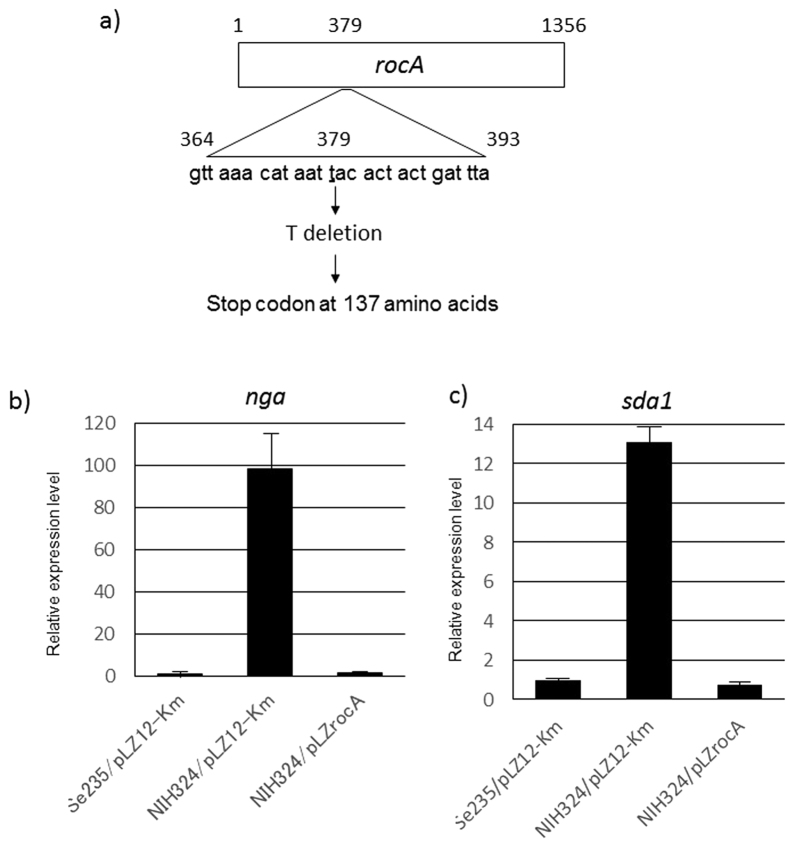
Mutation position of *rocA* and the transcriptional level of the *nga* and the *sda1* virulence genes by the *rocA* mutant. (**a**) The *rocA* gene sequence. A 1-bp (t) sequence was deleted in STSS isolate. Box indicates the *rocA* gene. The expression of the (**b**) *nga* and the (**c**) *sda1* virulence genes in Se235, NIH324, and NIH324 replaced with an intact *rocA* gene was analyzed by RT-PCR. Columns represent the relative mRNA expression levels of each gene from each strain. The expression level of Se235/pLZ12 strain is shown as 1. Values are represented as means ± SD (n = 4). Error bars show SD.

In the remaining two isolates, NIH324 and NIH388, the expression of all five virulence genes increased. We hypothesized that this was due to mutations in genes involved in regulation. Thus, we sequenced regulator genes in the two isolates and the non-STSS isolate Se235. Particularly, we sequenced 152 genes annotated as involved in regulation in the *S. pyogenes* MGAS5005 genome sequence (GenBank accession number CP000017). To note, the 152 regulator genes sequenced in Se235 were used as reference sequences. Compared to Se235, NIH388 was mutated in the *spy0367* (*scaR*) gene, and NIH324 was mutated in the *spy1318* (*rocA*) gene (Fig. 3a). Each intact gene derived from Se235 was inserted in pLZ12-Km to perform complementation tests. The increase of mRNA abundance of all five virulence genes in NIH324 was complemented, and thus reduced to levels similar to Se235, upon introduction of pLZrocA, which contained the *rocA* gene (Fig. 3b,c and data not shown). The increase of virulence gene expression in NIH388 was not complemented upon introduction of pLZscaR, which contained the *scaR* gene (data not shown). These results showed that enhanced virulence gene expression in NIH324 was due to a mutation in *rocA*. With regard to NIH388, increased virulence was not due to mutations in either gene. Two kinds of NIH388 strains were clinically isolated, one which formed mucoid colonies and the other non-mucoid colonies. The mucoid colony strain up regulated the expression of all virulence genes (Table 1), while the non-mucoid colony strain had comparable expression levels of virulence genes as Se235. We sequenced and compared the whole genomes of these two NIH388 strains. Although 11 putative SNPs were observed, these were not the causative agents of the two strains’ differential regulation of virulence (data not shown).

### Mutation of the *sic* promoter or the *rocA* gene is important in STSS pathogenesis of mice

**Figure 4:**
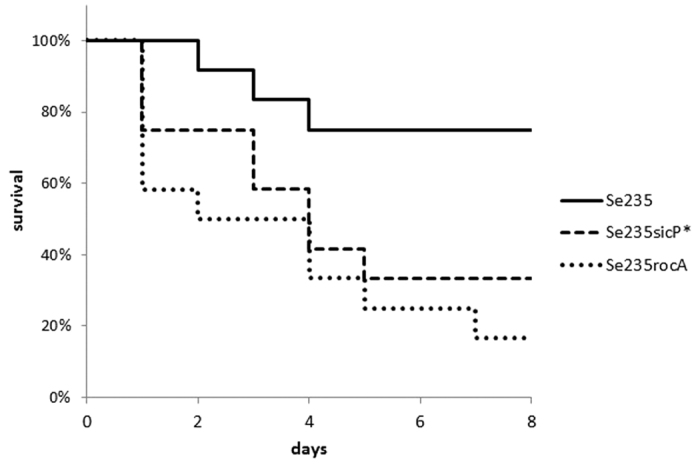
Mutation of *rocA* gene and *sic* promoter enhances the lethality of GAS mouse model. Survival curves of mice infected with either *rocA* (Se235rocA) or *sic* promoter (Se235sicP*) mutant strains or non-STSS Se235 strain. Mice were intraperitoneally inoculated with 4 × 10^7^ CFU of each GAS strain, and survival was observed for eight days post-infection. Mortality differences were statistically significant (*p* < 0.05) between Se235 and Se235sicP* and between Se235 and Se235rocA, as determined by a log-rank test. Survival curves were generated from three independent experiments using a total of 12 ddY mice for each strain.

In the 10 STSS *emm1* isolates, we found mutations in the *sic* promoter, the *csrR* promoter, or the *rocA* gene that conferred increased virulence gene expression. It is well-established that virulence is enhanced upon inhibition of *csrS/csrR* gene expression^6^. To elucidate the role of the *sic* promoter or *rocA* in STSS infection *in vivo*, we injected Se235 isolates with either the *rocA* or *sic* promoter mutation or Se235 alone in GAS model mice and compared the lethality between the two groups. The mutant strains showed significantly higher lethality than the Se235 strain (*p* < 0.05) (Fig. 4). This result suggested that *sic* promoter-mutated and *rocA*-mutated *emm1* strains isolated from STSS patients are more virulent than strains isolated from patients with non-invasive infections.

### Mutation frequency of the *rocA* gene and the *sic* promoter in STSS isolates

In this study, we showed that mutations in the *rocA* gene and the *sic* promoter of *emm1* clinical isolates from STSS patients caused lethality in mice, suggesting these mutations play important roles in the STSS pathogenesis. To evaluate the frequency of these in other *emm*-genotyped isolates, we sequenced the *rocA* gene and the *sic* promoter in STSS clinical isolates from sterile sites (164 isolates) and non-invasive clinical isolates from non-sterile sites (59 isolates)^3^.

SIC and DRS (distantly related to SIC) are related proteins present in only four (*emm1, emm12, emm55* and *emm57*) of the >200 GAS *emm* types^7^. We sequenced upstream regions of *sic* and *drs* in 103 *emm1* (73 STSS isolates, including six from this study, and 30 non-invasive isolates) and 12 *emm12* isolates (10 STSS isolates and 2 non-invasive isolates). There were no *emm55* and *emm57* isolates. In *emm1* isolates, a total of 10 STSS isolates (13.7%) had the *sic* promoter insertion mutation, which was not in any of the *emm1* non-invasive isolates. The position of mutation and the insertion sequence in the *sic* promoter were the same in all the mutated isolates. STSS *emm1* isolates had a *sic* promoter mutation at a significantly higher frequency than non-invasive isolates (*p* = 0.0329; Χ^2^ analysis). In *emm12* isolates, no mutations were observed in the putative *drs* promoter region. These results highlighted that the *sic* promoter mutation is unique to STSS *emm1* isolates. We sequenced the *rocA* gene in various *emm*-genotyped isolates in STSS isolates (164 isolates) and non-invasive isolates (59 isolates). A total of 30 STSS (18.3%) and 15 non-invasive (25.4%) isolates were mutated in the *rocA* gene. The position of this mutation was the same as previously described^8^,^9^. There was no significant difference in the mutation frequency between STSS isolates and non-invasive isolates (*p* = 0.242 by Χ^2^ analysis). Collectively, these results suggested that the *sic* promoter insertion mutations cause STSS rather than the *rocA* mutation.

**Figure 5:**
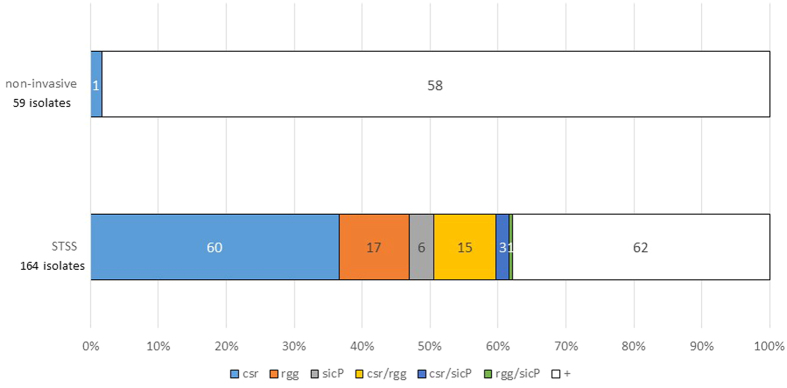
Mutation frequency in the *csr, rgg*, and *sic* promoters. The mutation frequency of *csr*, *rgg*, and *sic* promoter among *S. pyogenes* isolates from STSS (164 isolates) and non-invasive infections (59 isolates). STSS isolates (62.2%) had one or mutations in the *csrS/R*, *rgg*, and/or *sic* promoter, whereas isolates from patients with non-invasive disease had significantly fewer mutations in these genes (1.7%). ^+^indicated that all the genes were intact. The number in box indicated the number of isolates in each mutant. The number of STSS and non-invasive isolates in each *emm* genotype described below: *emm1* (STSS 73 isolates, non-invasive 30 isolates); *emm3* (26, 15); *emm4* (3, 2); *emm6* (1, 1); *emm11* (3, 2); *emm12* (10, 2); *emm18* (1, 0); *emm22* (7, 1); *emm28* (7, 2); *emm49* (5, 3); *emm53* (1, 0), *emm58* (2, 1); *emm59* (1, 0); *emm60* (1, 0); *emm77* (1, 0); *emm78* (1, 0); *emm81* (4, 0); *emm87* (4, 0); *emm89* (10, 0); *emm91* (1, 0); *emm112* (1, 0); *emm113* (1, 0).

In this study, 62.2% of the STSS isolates had mutations in the *csrS/R*, *rgg*, and/or *sic* promoter, whereas isolates from patients with non-invasive disease had significantly fewer mutations in these genes (1.7%) (Fig. 5).

## Discussion

In this study, we found new mutations in the *sic* promoter, the *csrR* promoter, or the *rocA* gene in STSS *emm1* isolates. We showed that the *sic* promoter and *rocA* mutations increased the expression of virulence gene(s) and enhanced lethality in the mouse model. Mutation of the *sic* promoter was found at a significantly higher frequency in STSS isolates than in the non-invasive isolates, whereas mutation of *rocA* did not show the same patter.

In our previous study, the frequency of mutations in the *csrS/R* and/or *rgg* genes in *emm* isolates other than *emm1* was 71.4%, whereas the frequency in *emm1* isolates was 39.7% (29/73); 44 isolates did not have mutation in these genes at all^3^. Specifically in this study, 62.2% of the STSS isolates had mutations in the *csrS/R*, *rgg*, and/or *sic* promoter, whereas isolates from patients with non-invasive disease had significantly fewer mutations in these genes (1.7%) (Fig. 5). Additionally, 50.7% (37/73) of *emm1* STSS isolates had mutations in the *csrS/csrR, rgg*, and/or the *sic* promoter. However, mutation frequency was still significantly lower in *emm1* STSS isolates than in the other *emm* genotypes (*p* = 0.0065 by Χ^2^ analysis). It is possible that additional mechanisms cause the upregulation of virulence genes in *emm1* STSS isolates.

STSS isolates had a significantly higher frequency of the *sic* promoter mutation than did non-invasive isolates. We showed that this mutant was important in the pathogenesis of invasive infection in the GAS mouse model. In our previous study, we found mutations in negative regulators that led to overproduction of a number of virulence factors^3^. The mutation in the *sic* promoter is different in that only one protein (the virulence factor SIC) was probably overexpressed, and this mutation led to the overexpression of SIC, implicating it as a crucial factor to STSS pathogenesis *in vivo*. Particularly, SIC protein is a secreted protein that interferes with complement and antibacterial proteins/peptides^10^,^11^,^12^; it is found in only two GAS *emm* genotypes, *emm1* and *emm57*^10^,^13^. About 50% of STSS is caused by *emm1* GAS in Japan^14^. Thus, we speculate that *emm1* isolates with this mutation are poised to be extremely virulent due to overexpression of this unique factor. The consensus sequence (ATTARA) for CsrR^15^ is found next to the *sic* promoter 6-bp insertion mutation. However, as the consensus sequence is not disrupted by the insertion, we do not know if CsrR binding was affected. Perez *et al*.^16^ reported 42 small regulatory RNAs in *emm1* isolates. The small RNA, SR1681917, is positioned in the upstream region of the *sic* gene^16^, and this small RNA may regulate *sic* gene expression. The *sic* gene may also be regulated by other small RNAs^17^. Structural changes in the RNA binding site of *sic* may have occurred due to the 6-bp insertion, and the small RNAs would not be able to bind, perhaps leading to upregulation of *sic*.

When the length of the spacer between the −35 and −10 elements of the *csrR* promoter was decreased from 17 bp to 16 bp, expression of the *csrR/csrS* operon also decreased. Recently, Rosinski-Chupin *et al*.^18^ reported on the consensus sequence of the sigma 70-dependent promoter in *S. agalactiae*. In this study, 17 bp was the most common spacer length between the −10 and −35 DNA elements. It is also well established that promoters with a 17-bp spacer yield higher levels of transcription than otherwise identical promoters with 16-bp spacers^19^,^20^,^21^. These studies further corroborate our findings that decrease of *csrR* gene transcription observed in *emm1* isolates was due to shorter spacer length.

The frequency of mutation in the *rocA* gene was not significant between STSS and non-invasive isolates. Lynskey *et al*.^8^ and Miller *et al*.^9^ reported that the RocA protein in *emm18* and *emm3* isolates is truncated and leads to the enhanced expression of virulence factors. In this study, RocA in an *emm18* isolate and many *emm3* isolates were truncated at the same position in both STSS isolates and non-invasive isolates. RocA in an *emm11* isolate and an *emm81* isolate was also truncated at the same position. While *rocA* mutations in *emm1* isolates may not have been significantly observed at a higher frequency in STSS vs. non-STSS isolates, such mutations may be important in establishing virulent clones in other *emm* genotypes, like *emm18* and *emm3*.

In conclusion, we have identified novel mutations that increased virulence gene(s) expression in STSS *emm1* isolates, specifically the *sic* promoter or the *rocA* gene. These mutations caused lethality in mice, and the significantly higher frequency of the *sic* promoter mutation in STSS *emm1* isolates implicates this mutation of utmost importance in the landscape of STSS onset and pathogenesis.

## Methods

### Ethics statement

This study complies with the guidelines of the declaration of Helsinki. This study protocol was approved by the institutional individual ethics committees for the use of human subjects (the National Institute of Infectious Diseases Ethic Review Board for Human Subjects) and the animal experiments (the National Institute of Infectious Diseases Animal Experiments Committee). Written informed consent was obtained from study participants. All clinical samples were stripped of personal identifiers not necessary for this study. All animal experiments were performed according to the Guide for animal experiments performed at National Institute of Infectious Diseases, Japan.

### Plasmids, bacterial strains, and culture conditions

The plasmids used in this study are described in Table 2. STSS criteria were based on those proposed by the Working Group on Severe Streptococcal Infections^22^. The clinical isolates from STSS and non-invasive (non-STSS) infections were collected during 1973–2008 as previously reported^3^. *Escherichia coli* DH5α was used as a host for plasmid construction and was grown in liquid of Luria-Bertani medium with shaking or on agar plates at 37 °C. GAS was cultured in Todd-Hewitt broth (Becton Dickinson, Tokyo, Japan) supplemented with 0.5% yeast extract (THY media) without agitation or on Columbia Agar supplemented with 5% sheep blood (Becton Dickinson). Cultures were grown at 37 °C in 5% CO_2_. When required, antibiotics were added to the medium at the following final concentrations: kanamycin (Km), 25 μg/mL for *E. coli* and 200 μg/mL for GAS; spectinomycin (Sp), 25 μg/mL for *E. coli* and GAS. The growth of GAS was turbidimetrically monitored at 600 nm using MiniPhoto 518R (Taitec, Tokyo, Japan).

### DNA sequencing

Nucleotide sequences were determined using automated sequencers, such as the Applied Biosystems 3130xl Genetic Analyzer (Applied Biosystems, Tokyo, Japan).

### Animals

Male 5–6-week-old outbred ddY mice were purchased from SLC (Shizuoka, Japan) and were maintained in a specific pathogen-free (SPF) condition. All animal experiments were performed according to the guidelines of the Ethics Review Committee of Animal Experiments of the National Institute of Infectious Diseases, Japan.

### Construction of the *sic*-*phoZF* transcriptional fusion plasmid

To construct plasmids that could measure *sic* transcriptional levels, we used the pABG5 plasmid^23^ that has the *phoZF* reporter gene. When expressed, this reporter gene will produce alkaline phosphatase that is secreted from cells and can easily be measured. In the engineering of this construct, we deleted the promoter region of the *phoZF* gene but not the Shine-Dalgarno (SD) region of pABG5, resulting in pABG0. We amplified the *sic* promoter region of STSS and non-STSS isolates via PCR using the primers: sic-up1 (5′-GGGGATCCATTAGCGAAACAAGCTGAAG-3′) and sic-up4 (5′-GGGAATTCCAGTCATCTCCAGACCAGTC-3′). PCR products were digested with *Bam*H1 and *Eco*R1 in order to clone into the *Bam*HI-*Eco*RI site upstream of the *phoZF* SD region of pABG0, resulting in the fusion plasmids pABGsic(Se) and pABGsic(270).

### Construction of the *csrR*-*phoZF* transcriptional fusion plasmid

In order to engineer fusion plasmids capable of measuring *csrR* promoter activity, we amplified the *csrR* promoter region of STSS and non-STSS isolates by PCR with the primers: covP1-Bm (5′-GGGGATCCCTTGCAAGGGTTGTTTGATG-3′) and covPR3-Ec (5′-GGGAATTCCAAGAGAAACGAATCTAGCC-3′). PCR products were digested with *Bam*H1 and *Eco*R1 in order to clone into the *Bam*HI-*Eco*RI site upstream of pABG0, resulting in the fusion plasmids pABGcsr(17spacer) and pABGcsr(16spacer).

### Construction of *scaR* and *rocA* complementation plasmids

To map potential mutations in the *scaR* and *rocA* transcriptional regulator genes found in STSS isolates, these genes were amplified via PCR from the non-STSS isolate Se235. The *scaR* gene was amplified from Se235 using the primers: scaRF-Bm (5′-GGGGATCCCTTTCCCATCATTTCTCTCC-3′) and scaRR-Ec (5′-GGGAATTCAAGTCAAAGGCTTAAAAATGG-3′). The *rocA* gene was amplified from Se235 using the primers: rocAop1 (5′-GGGCCATGGGAAGGAGAAGGATAAATGTTAG-3′) and rocA6-Ec (5′-GGGAATTCGGGACTATTGTCTCAGACTC-3′). The resulting PCR products were inserted into pLZ12-Km^24^, yielding pLZscaR and pLZrocA, respectively.

### Construction of deletion or insertion mutants

#### Construction of the *rocA* mutant

To create a *rocA* deletion mutant, four DNA fragments were amplified via PCR. A DNA fragment containing the 5′ end of *rocA* and the adjacent upstream chromosomal DNA was amplified from the NIH324 STSS *emm1* isolate using the primers: rocA-del1 (5′-TCGACGTCCGGATCCGGGAAGGTCAAGTCTGTGCGGG-3′) and rocA-del2 (5′-ACGAAAATCAAGCTTGAGAAGGAGAAGGATAAATG-3′). A fragment containing the 3′ end of *rocA* and the adjacent downstream chromosomal DNA was amplified from the NIH324 using the primers: rocA-del3 (5′-AATGGTGGAAACACTCAAAATCAACTTAAGAGTC-3′) and rocA-del4 (5′-GCCTTTTTTACGCGTCTTAACATCATTAGCAACACC-3′). A DNA fragment containing pFW12 (pFW12-PCR) was amplified using the primers: pFW12-PCR1 (5′-GGATCCGGACGTCGACGGCCGTA-3′) and pFW12-PCR2 (5′-ACGCGTAAAAAAGGCCCACAAAAGTGGG-3′). A DNA fragment containing *aad9*, spectinomycin resistant gene, (spc-PCR) was amplified from the pFW12^25^ using the primers: spc3 (5′-AAGCTTGATTTTCGTTCGTG-3′) and spc4 (5′-AGTGTTTCCACCATTTTTTC-3′). These four PCR products were circularized using the In-Fusion HD cloning kit (Takara Bio, Shiga, Japan), following the manufacturer’s instructions, in order to create the plasmid pFWrocA. This plasmid was then introduced into Se235 by electroporation, and transformants were selected on Columbia Agar plate supplemented with 5% sheep blood containing spectinomysin. The replacement of the native *rocA* gene by the *rocA*-deleted mutant allele was verified by PCR, and the resultant strain was named Se235rocA.

#### Construction of the *sic* promoter disruption mutant

To construct *sic* promoter disruption mutants, two DNA fragments were amplified via PCR. A DNA fragment containing the upstream region of the *sic* promoter was amplified from the NIH270 *emm1* STSS isolate chromosomal DNA using the primers: sicP-ins1 (5′-TCGACGTCCGGATCCGTTAGAAACGATTACTAGAG-3′) and sicP-ins2 (5′-ACGAAAATCAAGCTTCAGTATTACAAAGTGATAG-3′); a fragment containing the *sic* promoter and the adjacent downstream chromosomal DNA was amplified from NIH270 was amplified using the primers: sicP-ins3 (5′-AATGGTGGAAACACTCTGAGTGAACATCAAGAGAG-3′) and sicP-ins4 (5′-GCCTTTTTTACGCGTCGTCTGACCAGCCACCATAC-3′). These two respective PCR products, pFW12-PCR and spc-PCR, were circularized using the In-Fusion HD cloning kit in order to create the plasmid pFWsicP*. This plasmid was then introduced into the non-STSS strain Se235 by electroporation, and transformants were selected as described above. The replacement of the native *sic* promoter gene by the *sic* promoter disruption mutant was verified by PCR and sequencing, and the resultant strain was named Se235sicP*.

### *phoZF* alkaline phosphatase activity assay

The reporter gene *phoZF* used in this study encodes a chimeric protein consisting of both the N-terminal domain of protein F and the C-terminal domain of *Enterococcus faecalis* alkaline phosphatase (*phoZ*). PhoZF is secreted from the cell and detected in the supernatant. Alkaline phosphatase activity was measured as previously described^26^.

### Quantitative RT-PCR analysis

To examine the expression of virulence genes in *emm1*-genotyped isolates, total RNA was extracted from 44 *emm1*-genotyped STSS isolates and 3 *emm1*-genotyped non-invasive isolates (negative controls), and we performed quantitative RT-PCR to assess the transcriptional abundance of five virulence genes, *scpA, sic, sda1, nga*, and *ska*, known to be elevated in invasive strains^4^. Briefly, GAS was grown to late-log phase (OD600 = 0.8–1.0) at 37 °C in a 5% CO_2_ atmosphere, and total RNA was extracted from bacterial cells using the RNeasy Protect Bacteria Mini Kit (QIAGEN), according to the manufacturer’s instructions. Complementary DNA synthesis was performed with the PrimeScript RT reagent Kit (Perfect Real Time) (Takara Bio), following the manufacturer’s instructions. Transcript levels were determined using the ABI PRISM Sequence Detection System 7000 (Applied BioSystems) and Premix Ex Taq (Perfect Real Time) (Takara Bio). For real-time amplification, the template was equivalent to 5 ng of total RNA. Measurements were performed in triplicate; a reverse-transcription-negative blank of each sample and a no-template blank served as negative controls. *gyrA* was used as an internal control. Primers and probes were described Supplementary Table 1.

### Complete-genome comparisons

The whole genome of STSS *emm1* strain NIH388 was sequenced by paired-end sequencing on an Illumina Hiseq2000 sequencing system (Cosmo-Bio Co., Ltd., Tokyo, Japan), and comparison was performed by a data mining service (Filgen Co., Ltd., Aichi, Japan). The reference genome sequence used was *S. pyogenes* 476 (GenBank accession No. AP012491).

### GAS infection in a mouse model

A total of 4 × 10^7^ CFU of GAS suspended in 0.5 mL PBS was injected intraperitoneally into five- to six-week-old ddY outbred male mice (12 mice/GAS strain). The number of mice that survived was compared statistically using the Kaplan-Meier log-rank test.

## Additional Information

**How to cite this article**: Ikebe, T. *et al*. Spontaneous mutations in *Streptococcus pyogenes* isolates from streptococcal toxic shock syndrome patients play roles in virulence. *Sci. Rep*. **6**, 28761; doi: 10.1038/srep28761 (2016).

## Supplementary Material

Supplementary Information

## Figures and Tables

**Table 1:** Virulence gene expression and mutation positions of *emm1* STSS isolates.

Strain number	Gene^a)^	Gene^a)^	Gene^a)^	Gene^a)^	Gene^a)^	mutation
	*sic*	*scpA*	*sda1*	*nga*	*ska*	
135	+					*sic* promoter
150	+					*sic* promoter
185	+					*sic* promoter
270	+					*sic* promoter
392	+					*sic* promoter
436	+					*sic* promoter
342	+	+	+	+	+	*csrR* promoter
344	+	+	+	+	+	*csrR* promoter
324	+	+	+	+	+	*rocA*
388	+	+	+	+	+	unknown

a) + indicates >10-fold increase of gene expression.

**Table 2:** Plasmids used in this study.

Plasmid name	Relevant characteristics	References
pABG5	promoter prove vector carrying *rofA* promoter, Km^r^	23
pABG0	Deleted *rofA* promoter from pABG5	26
pABGsic(Se)	pABG0 containing *sic* promoter derived from Se235	This study
pABGsic(270)	pABG0 containing *sic* promoter derived from NIH270	This study
pABGcsr(17spacer)	pABG0 containing *csr* promoter derived from Se235	This study
pABGcsr(16spacer)	pABG0 containing *csr* promoter derived from NIH344	This study
pLZ12-Km	*E. coli/Streptococcus* sp. shuttle vector, Km^r^	24
pLZscaR	pLZ12-Km containing *scaR* gene derived from Se235	This study
pLZrocA	pLZ12-Km containing *rocA* gene derived from Se235	This study
pFW12	*E. coli* cloning vector, Sp^r^	25
pFWrocA	5′ and 3′ flanking region of *rocA* in pFW12	This study
pFWsicP*	5′ and 3′ flanking region of *sic* promoter in pFW12	This study
