# Supplementary material for: Spontaneous mutations in Streptococcus pyogenes isolates from streptococcal toxic shock syndrome patients play roles in virulence
Source: Sci Rep. 2016 Jun 28;6:28761. doi: 10.1038/srep28761 (PMC4923885; doi:10.1038/srep28761)
Supplement: Supplementary Information [file srep28761-s1.doc]

**Supplementary information**

Spontaneous mutations in *Streptococcus pyogenes* isolates from streptococcal toxic shock syndrome patients play roles in virulence

Tadayoshi Ikebe, Takayuki Matsumura, Hisako Nihonmatsu, Hitomi Ohya, Rumi Okuno, Chieko Mitsui, Ryuji Kawahara, Mitsuhiro Kameyama, Mari Sasaki, Naomi Shimada, Manabu Ato, and Makoto Ohnishi

**Ikebe et al. Supplementary Table 1**

**Supplementary Table 1. Primers used for RT-PCR**

| gene | Primer | Sequence (5′–3′) |
| --- | --- | --- |
| *sic* | sic-RTP1 | GAGGACACCCCTCCAAGTGA |
|  | sic-RTP2 | TCTTGTGGATTTTTTTGAGGAGTATG |
| *scpA* | scpA-RTP1 | GACAGCTGACGGCAATATTAAGC |
|  | scpA-RTP2 | TGTTAGCCACTGACGACAAAATATC |
| *sda1* | sda1RTP1 | TGAATCGGGCACTATTTCAAATAA |
|  | sda1RTP2 | CAGTTGCGCTTTACCTTCAACA |
| *nga* | nga-RTP1 | ACGTACGCTGTTAGTGGCAAAG |
|  | nga-RTP2 | AGGAAGTTGCGTTAGCTTCCAT |
| *ska* | ska1RTP1 | AACAAAACCCACCCAGGCTAT |
|  | ska1RTP2 | TCGTACGGAAAATGTCATTGTCA |
| *gyrA* | gyrA-RTP1 | CATGAGTGTCATTGTGGCAAGA |
|  | gyrA-RTP2 | CGACGATGCACAGGTTTCAG |
| TaqMan probe |  |  |
| *sic* | sic-TMP1 | CCTCGTGTGCCAGAAAAACCGCA |
| *scpA* | scpA-TMP1 | AGATATTGCAGCACCCGGCCA |
| *sda1* | sda1-TMP1 | TGGAGTATCGAGCAACACCCCAATTATTACC |
| *nga* | nga-TMP1 | TAAAAAAAGCGATGTCAAATATGAAACGACCAAAGT |
| *ska* | ska1TMP1 | CGATTTATGAACGTGACTCCTCAATCGTCA |
| *gyrA* | gyrA-TMP1 | CTTTGCCAGATGTGCGTGATG |
